# Homing of Antigen-Presenting Cells in Head Kidney and Spleen – Salmon Head Kidney Hosts Diverse APC Types

**DOI:** 10.3389/fimmu.2013.00137

**Published:** 2013-06-06

**Authors:** Dimitar B. Iliev, Hanna Thim, Leidy Lagos, Randi Olsen, Jorunn B. Jørgensen

**Affiliations:** ^1^Norwegian College of Fisheries Science, University of Tromsø, Tromsø, Norway; ^2^Department of Medical Biology, University of Tromsø, Tromsø, Norway

**Keywords:** MHCII, APC, granulocytes, Atlantic salmon, CpG oligonucleotides, IL-1β, TNF, autophagy

## Abstract

Lymph nodes and spleen are major organs where mammalian antigen-presenting cells (APCs) initiate and orchestrate Ag-specific immune responses. Unlike mammals, teleosts lack lymph nodes and an interesting question is whether alternative organs may serve as sites for antigen presentation in teleosts. In the current study, fluorescent ovalbumin (Ova) and CpG oligonucleotides (ODNs) injected intra-abdominally were detected in significant numbers of salmon head kidney (HK) MHCII+ cells over a period of 2 weeks while in spleen the percentage of these was transient and declined from day 1 post injection. *In vitro* studies further shed light on the properties of the diverse MHCII+ cell types found in HK. The ultrastructure of a subpopulation of MHCII+ cells with a high capacity to endocytose and process Ova indicated that these were able to perform constitutive macropinocytosis. Upon stimulation with CpG ODNs these cells upregulated CD86 and gave very high levels of TNF mRNA indicating that these are professional APCs, related to macrophages and dendritic cells (DCs). A subpopulation of HK granulocytes expressed high levels of surface MHCII and upon CpG stimulation upregulated most of the tested APC marker genes. Although these granulocytes expressed TNF weakly, they had relatively high basal levels of IL-1β mRNA and the CpG stimulation upregulated IL-1β, along with its signaling and decoy receptors, to the highest levels as compared to other HK cell types. Interestingly, the high expression of IL-1β mRNA in the granulocytes correlated with a high autophagy flux as demonstrated by LC3-II conversion. Autophagy has recently been found to be implicated in IL-1β processing and secretion and the presented data suggests that granulocytes of salmon, and perhaps other teleost species, may serve as a valuable model to study the involvement of autophagy in regulation of the vertebrate immune response.

## Introduction

Antigen-presenting cells (APCs) are specialized to take up, process and present protein antigens (Ag) associated with MHCII molecules to Ag-specific T-cells. Professional APCs include dendritic cells (DCs), macrophages, and B-cells of which the DCs are distinguished by their high co-stimulatory capacity (Hamilos, 1989; Thery and Amigorena, 2001). APCs are distributed throughout the organism, including the peripheral tissues where they capture inbound Ags derived from potentially pathogenic agents. In order to achieve full co-stimulatory capacity, APCs need to be activated through innate immune receptors such as toll-like receptors (TLRs) which recognize conserved molecular structures produced by microorganisms and viruses (Thery and Amigorena, 2001). In mammals, exposure of DCs to these pathogen-associated molecular patterns (PAMPs) may trigger a process of maturation during which the antigen-loaded DCs migrate toward the secondary immune organs, upregulate MHCII-associated Ag peptides and co-stimulatory molecules on their surface and downregulate their capacity to endocytose Ag (Kaisho and Akira, 2003). In mammals, the major organs where maturing DCs migrate and which provide proper environment for Ag presentation are the lymph nodes and the spleen.

The adaptive immune system has evolved at the level of early jawed vertebrates and most of its elements described in mammals, such as Ag-specific B- and, T-cell receptors and antibodies are also found in teleosts (Pancer and Cooper, 2006). In addition, a T-cell-dependent Ag response has been described in teleosts (Miller et al., 1986) and PAMPs, such as CpG oligonucleotides (ODNs), have shown a potential to induce maturation of salmon APCs *in vitro* (Iliev et al., 2010). Cells resembling mammalian DCs have been described in fish (Lovy et al., 2009; Aghaallaei et al., 2010; Lugo-Villarino et al., 2010; Bassity and Clark, 2012) and authors have suggested that the melanomacrophage centers which are found in the spleen and the head kidney (HK) (anterior kidney, pronephros) of different teleosts (Tsujii and Seno, 1990) may serve as sites for Ag presentation (Agius and Roberts, 2003). Still, there are considerable differences between the mammalian and the teleost immune systems – for example, absence of lymph nodes and classical Ig class switch in teleosts. Therefore, more detailed knowledge about the phenotype and the function of piscine APCs will help gain insight into the evolution of the vertebrate adaptive immune system and will provide valuable information for development and optimization of immunotherapies for aquaculture use.

In an earlier study, we found that Atlantic salmon HK hosts distinct MHCII+ cell types including MHCII+ leukocytes which endocytosed large amounts of dextran and a population of granular cells with lower capacity to take up dextran but with high levels of surface MHCII as shown by staining with an antibody specific for the MHCII beta chain (Iliev et al., 2010). Additionally, the HK harbored MHCII/Ig double-positive cells, resembling B-cells.

In light of these findings, the current study has been intended to further characterize salmon APCs in regard to their ability to migrate to HK and spleen following *in vivo* uptake of Ag, their morphology, and their potential to express immune genes including APC markers, cytokines, and cytokine receptors. Fluorescent ovalbumin (Ova-FITC) injected in the abdominal cavity was observed both in HK and spleen leukocytes 1 day post injection, while at latter time points (5 and 14 days) it was found exclusively in MHCII+/IgM− HK cells. When HK cells were stimulated *in vitro*, distinct gene expression profiles were detected in different HK MHCII+ cell types. The cells which endocytosed high amounts of Ova expressed the highest levels of CD86 and TNF mRNA suggesting that these are maturing APCs. On the other hand, a population of cells with morphology of polymorphonuclear granulocytes which expressed high levels of surface MHCII and did not take up significant amounts of Ova, upregulated very high levels of IL-1β mRNA along with APC marker genes including CD83, CD40, and B7-H1 but they expressed relatively low levels of CD86. Autophagy has recently been found to be involved in production and secretion of IL-1β (Dupont et al., 2011) and autophagy flux analysis using sorted cell populations showed that the intensity of the process was highest in the HK granulocytes suggesting that these cells might be specialized in production and secretion of this cytokine.

## Materials and Methods

### Fish

Atlantic salmon (*Salmo salar*) strain AquaGen standard (Aqua Gen, Kyrksæterøra, Norway) were obtained from the Tromsø Aquaculture Research Station (Tromsø, Norway). The fish were kept at about 10 °C in tanks supplied with running filtered water and were fed on commercial, dry food (Skretting, Stavanger, Norway). All experiments were approved by the national committee for animal experimentation (Forsøksdyrutvalget, Norway) and performed according to its guidelines.

### Reagents

Phosphorothioate-modified CpG-B ODNs (5′-TCGTCGTTTTGTCGTTTTGTCGTT-3′) were purchased from Thermo Scientific. The antiserum against the β-chain of salmon MHCII was previously described (Iliev et al., 2010). The antibody against salmonid IgM was obtained from CedarLane Labs, ON, Canada. A secondary goat anti-rabbit antibody conjugated with AlexaFluor546, Ova-Alexa647, DQ Ova, and LysoSensor™ Green DND-189 were purchased from Life technologies. Goat anti-rabbit-horseradish peroxidase antibody was obtained from Santa Cruz Biotechnology and the eEF2 polyclonal antibody was purchased from Cell Signaling Technology. Polyclonal LC3 antibody, dextran-Cascade Blue, Ova-FITC, and May Grünwald–Giemsa reagents were obtained from Sigma.

### Isolation of leukocytes from Atlantic salmon

Three groups of 15 individuals of ~100 g were injected intra-abdominally with 100 μl of PBS, 500 μg of Ova-FITC, or with, 500 μg of Ova-FITC + 50 μg of CpG-Cy5. HK and spleen leukocytes were isolated 1, 5, and 14 days post injection as described (Iliev et al., 2010). Briefly, the HK and the spleen tissues were passed through 100-μm pore size cell strainers (Falcon) in L-15 medium containing penicillin (10 U/ml), streptomycin (10 μg/ml), 2% fetal bovine serum (FBS), and heparin (20 U/ml). The resulting suspension was placed on a 25/54% discontinuous Percoll gradient and centrifuged at 400 × g for 40 min at 4 °C. The cells at the interface were collected and washed twice in L-15 medium before further use. For the *in vitro* experiments, the density of the leukocyte suspensions was adjusted to 7 × 10^6^ cells/ml and the cells were further incubated in 24-well plates in L-15, 5% FBS.

### Flow cytometry

Cells were pelleted at 4 °C and the staining was performed on ice. The cells were washed with ice-cold PBS and incubated simultaneously with MHCIIβ rabbit antiserum (1000-fold dilution) and salmon anti-IgM FITC-conjugated monoclonal antibody (200-fold dilution) for 1 h in PBS, 5% FBS on ice. The secondary Alexa546 goat anti-rabbit antibody was diluted to 1 μg/ml in PBS, 5% FBS and the cells were incubated for 30 min on ice prior to washing with PBS. For the *in vitro* endocytosis assays, HK leukocytes were incubated with 10 μg/ml of Ova-FITC or Ova-Alexa647, 150 μg/ml of dextran-Cascade blue and 2 μM CpG-Cy5 ODNs for 1 h. Prior to sorting, the whole HK leukocyte population was stimulated for 24 h with 2 μM CpG ODNs followed by 1 h incubation with 10 μg/ml of or Ova-Alexa647 prior to staining as described above. The cells were analyzed and sorted using FACSAria (Becton Dickinson).

To analyze the Ag-processing capacity of APCs found in salmon HK, cells were incubated with 5 μg/ml of Ova-DQ for 4 h. After removal of non-adherent cells, the adherent ones were detached and harvested using a 10 min treatment with PBS, 1 mM EDTA. The adherent and the non-adherent cells were stained for surface MHCII and analyzed separately with flow analysis. Ova-DQ proteolysis was detected as green fluorescence.

### Real-time PCR (RT-PCR) analysis

RNA from sorted cells was isolated using RNeasy Mini Kit (Qiagen). On-column DNase digestion was performed using RNase-Free DNase set (Qiagen, Hilden, Germany). For each sample 70 ng total RNA was reverse transcribed using the TaqMan Reverse Transcription Reagents kit (Applied Biosystems). The real-time PCR (RT-PCR) reactions were prepared with Power SYBR ^®^Green PCR Master Mix or TaqMan ^®^Fast Universal PCR Master Mix (Applied Biosystems). The type of the reaction and the primer and the probe sequences are listed in Table 1. The reactions were run in duplicate and included 5 μl of fivefold diluted cDNA. The reaction protocol and the data analysis have been previously described (Iliev et al., 2010). EF1aB expression was used as endogenous control and the data is presented as fold difference values as compared to the non-stimulated MHC−/Ig−/Ova−sample.

**Table 1:** **Sequences of the primers used in the PCR analysis**.

B7-H1	SYBR	Fwd	ACATGTGTCCAGGCTGAGGATCAA
	green	Rev	ATTGTGGCAAGAGGATAGCCCTCA
TNF decoy	SYBR	Fwd	AGCATTGCACAAAGGACCGCAA
receptor 3	green	Rev	ACACCTTCTGCGCCTTGAACAT
(DCR3)			
TNFRSF11B	SYBR	Fwd	ACGGGCCAGTTACTCACCTGTAAT
Osteoprotegerin	green	Rev	TGAGAACCGAGCATTCCTCCTTGA
(OPG)			
CD40	SYBR	Fwd	ATGCCATGCCAAGAGGGTGAAT
	green	Rev	ATTTGCATGGGCTGAGGCTTGT
TNF2	SYBR	Fwd	TGCTGGCAATGCAAAAGTAG
	green	Rev	AGCCTGGCTGTAAACGAAGA
CD86	SYBR	Fwd	ACTTCACACTCGATTACGGCTGCT
	green	Rev	AGCAGGAATAAGGTGACACACCGA
IL1R1	SYBR	Fwd	AATGCTACTGAGAGCCATGCTGGA
	green	Rev	ACTTTGAGCTGAGTGCTGTGGGTA
IL1R2	SYBR	Fwd	AGCGAGATCACTTGGGAGGTGTTT
	green	Rev	AAGTGTGTCACTCGAAACCAGGGA
IL-18	SYBR	Fwd	ATGACATTGACAGGCCCAGAGGAA
	green	Rev	GTTGCTCCAGTGGTTTGGCAGAAA
IL-15	SYBR	Fwd	CGTTTATTGGAGCGCAGGACAAAG
	green	Rev	CATGAGTTTCAGCAGCACCAGCAA
CD208	SYBR	Fwd	CAACCCTGAGCCCACCGAGC
	green	Rev	ATGCATGGCTTGCCTGCGGT
IL-10	SYBR	Fwd	ACTCCGCACATCCTTCTCCACCA
	green	Rev	TCATGGCGGTGGGCAACACC
CCR6	SYBR	Fwd	ACGCTGCTGCGTGCCAAGAA
	green	Rev	GCGCAGCGTTGTAGGGCAGA
CD83	Taqman	Fwd	GTGGCGGCATTGCTGATATT
		Rev	CTTGTGGATACTTCTTACTCCTTTGCA
		Probe	CACCATCAGCTATGTCATCC
IL-1β1	Taqman	Fwd	GCTGGAGAGTGCTGTGGAAGA
		Rev	TGCTTCCCTCCTGCTCGTAG
		Probe	TTGGAGTTGGAGTCGGCGCCC
IFNal	Taqman	Fwd	CCTTTCCCTGCTGGACCA
		Rev	TGTCTGTAAAGGGATGTTGGGAAAA
		Probe	CTTTGTGATATCTCCTCCCATC
IFNγ	Taqman	Fwd	AAGGGCTGTGATGTGTTTCTG
		Rev	TGTACTGAGCGGCATTACTCC
		Probe	TTGATGGGCTGGATGACTTTAGGA
Membrane	Taqman	Fwd	CCTACAAGAGGGAGACCGA
IgM		Rev	GATGAAGGTGAAGGCTGTTTT
(mlgM)		Probe	TGACTGACTGTCCATGCAGCAACACC
Secreted	Taqman	Fwd	CTACAAGAGGGAGACCGGAG
IgM		Rev	AGGGTCACCGTATTATCACTAGTTT
(slgM)		Probe	TCCACAGCGTCCATCTGTCTTTC4
PAX5	Taqman	Fwd	CCACTGCCAGGTCGAGA
		Rev	GTCAGCGAGGAGGTGGAGTA
		Probe	CCCCGGCTATCCACCACACG
EF1aB	Taqman	Fwd	TGCCCCTCCAGGATGTCTAC
		Rev	CACGGCCCACAGGTACTG
		Probe	AAATCGGCGGTATTGG

### Confocal microscopy

Head kidney cells from fish injected with Ova-FITC and CpG-Cy5 attached to 15 mm coverslips were washed, with PBS and fixed for 15 min with 4% formaldehyde. Following permeabilization using 0.3% Triton X-100 for 15 min at room temperature and blocking in PBS, 5% FBS for 30 min, the cells were incubated consecutively with 1:2000 dilution of MHCIIβ antiserum and a secondary Alexa546-conjugated antibody. The coverslips were mounted in glycerol, 1% DABCO. Images were collected with a Zeiss Axiovert 200 microscope with a 40×, Apochroma objective, equipped with an LSM510-META confocal module using the LSM5 software version 3.2 (Carl Zeiss Inc.).

For *in vitro* Ag uptake and sub-cellular localization, adherent HK mononuclear phagocytes were isolated as previously described (Iliev et al., 2010). To visualize endolysosomes, cells cultured in Lab-Tek™ Chambered Coverglass slides (Nunc) were incubated with 1 μM LysoSensor™ Green DND-189 (Life technologies) for 30 min, washed and incubated in L-15, 5% FBS with 10 μg/ml of Ova-Alexa647. Images of live cells were taken as described above.

### Western blot

Samples from sorted cells (1 × 10^5^ per sample) were pelleted (1500 g, 5 min), lyzed in LDS sample buffer (Invitrogen) and run on NuPAGE Novex Bis-Tris 12% gel (Invitrogen). The proteins were transferred to PVDF membranes, blocked with 5% dry milk and incubated consecutively with a 1:1000 dilution of LC3 antibody and a 1:10000 dilution of a goat anti-rabbit-horseradish peroxidase antibody. Stripping of the membranes was performed in 0.2 M NaOH for 10 min followed by washing, blocking, and reprobing with 1:1000 dilution of an eEF2 polyclonal antibody.

### Morphology of sorted HK cells – transmission electron microscopy analysis

Sorted HK subpopulations were pelleted in a microcentrifuge (1500 *g*, 5 min) and fixed in 8% formaldehyde. Cells were washed in 0.1% cacodylate buffer (pH 7.2), post fixed in mixture of 2% osmium tetroxide/1.5% potassium ferrocyanide in 0.1% cacodylate for 30 min. Staining was performed with 1% tannic acid and 1% uranyl acetate, followed by dehydration in a graded series of ethanol (70%, 90%, 96%, 2 × 100%). The cells were treated with acetonitrile as an intermediate step before infiltration with an Epon substitute (AGAR 100 resin, Agar Scientific, Stansted, England) and polymerized at 60 °C overnight. Ultrathin sections (70 nm) were made using a Leica Ultracut S Ultramicrotome (Vienna, Austria) with a Diatome diamond knife (Biel, Switzerland). The sections were mounted on carbon coated formvar films on copper grids and contrasted with 5% uranyl acetate for 8 min and Reynolds lead citrate for 5 min. Micrographs were taken on a Jeol 1010 JSM (Tokyo, Japan) with a Morada 11 Mpixels digital camera (Olympus).

### Immunolabeling for electron microscopy

Sorted HK subpopulations were pelleted in a microcentrifuge (1500 *g*, 5 min) and fixed in 4% formaldehyde in 200 mM HEPES buffer, pH 7.5, for 1 h. The samples were prepared for immunolabeling according to standard methods. Ultrathin cryosections cut on a Leica EMUC6 Ultramicrotome with an Ultracut S in a Leica FCS cryochamber using a diamond knife (Diatome Ltd., Bienne, Switzerland) and mounted on Formvar-coated EM-grids. Immunocytochemical labeling was performed as described (Tokuyasu, 1986; Webster and Webster, 2007). The Anti-LC3 antibody was used at 1:1000 dilution and was detected by protein A-gold complexes. The dried sections were examined in a JEOL JEM 1010 transmission electron microscope (JEOL, Tokyo, Japan) operating at 80 kV. Control experiments were routinely included in parallel by omission of the primary antibodies.

### Data analysis

Data was analyzed with one-way and two-way ANOVA, as indicated, followed by Tukey post-tests. Statistical analyses were performed using the GraphPad Prism6 software. The value of *p* < 0.05 was considered to be significant. Only groups with *n* > 3 were included in the analysis.

## Results

### Soluble antigen and CpGs injected in the abdominal cavity of Atlantic salmon accumulate predominately in HK MHCII+ cells

**Figure 1:**
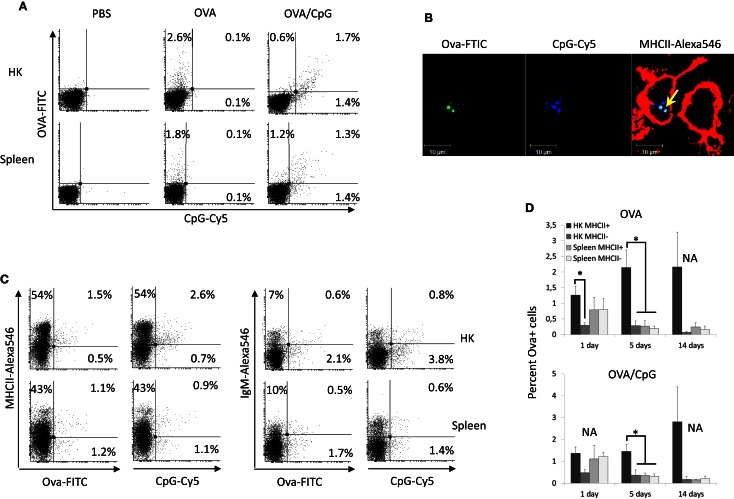
**Fluorescent Ova-FITC and CpG-Cy5 ODNs injected in the abdominal cavity of Atlantic salmon accumulate predominately in HK MHCII+ cells**. The fish were injected with Ova-FITC alone or in combination with CpG-Cy5 and their accumulation in head kidney (HK) and spleen leukocytes was analyzed with flow cytometry after 1, 5, and 14 days. The controls were injected with the same volume of PBS. **(A)** 24 h post injection approximately equal percentage of Ova+ and CpG+ cells was observed in HK and spleen and there was good correlation between the uptake of Ova and CpG ODNs. **(B)** HK cells from Ova/CpG-injected fish were stained for MHCII and were analyzed with confocal microscopy. The arrow indicates the colocalization between Ova and CpGs in endosomal compartments of MHCII+ cells. **(C)** Ova and CpG ODNs accumulate mostly in MHCII+/IgM− cells in HK. MHCII- and IgM-stained cells were analyzed with flow cytometry. The dot plots show the correlation between the surface expression of MHCII and IgM and the uptake of Ova and CpG ODNs in HK and spleen leukocytes isolated 24 h after injection. **(D)** The percentage of Ova+ cells in spleen declines sharply between 1 and 5 days post injection whereas in HK it remains high for up to 14 days. The histograms show the mean percentage of Ova+ cells in MHCII± cells in both the Ova and the Ova/CpG groups as determined by flow cytometry (*n* varies between 2 and 5), Statistical analysis was performed on samples taken on day 1 and day 5 since the number of replicates in all of these samples was >3, *P < 0.05.

To analyze the Ova distribution *in vivo*, salmon (~100 g) were injected intra-abdominally with 500 μg of Ova-FITC or with 500 μg of Ova-FITC mixed with 50 μg of CpG-Cy5. Control fish were injected with the same volume (100 μl) of PBS. HK and spleen leukocytes were isolated after 1, 5, and 14 days and following staining for surface MHCII and IgM were analyzed using flow cytometry. The results shown in Figure 1A demonstrate that Ova and CpGs are detected in both spleen and HK cells 1 day post injection. The percentage of Ova+ and CpG+ cells in HK and spleen were approximately equal at this time point (~2% in both organs). Since, considerably more leukocytes were isolated from HK, the absolute number of Ova+ cells was greater in this organ as compared to spleen. Although there was relatively more CpG+ than Ova+ cells in HK, there was a relatively good correlation in the uptake of the two substrates. It seems that the higher percentage of CpG+ cells might be due to a higher intensity of the CpG-Cy5 fluorescence as compared to that emitted by Ova-FITC. Confocal microscopy analysis revealed that Ova-FITC and CpG-Cy5 colocalized in intracellular vesicles in MHCII+ cells from HK harvested 24 h post injection (Figure 1B). These cells had a macrophage-like morphology as revealed by the presence of long and branching pseudopodia.

The dot plots presented in Figure 1C show cells isolated 1 day post injection. Most of the Ova+ and CpG+ HK cells expressed surface MHCII but not IgM. In contrast, in spleen an approximately equal percentage of the Ova+ and the CpG+ cells were MHCII− and MHCII+, and most of these were IgM−. The histograms in Figure 1D show the average percentage of the Ova+ cells in the Ova-only and the Ova/CpG-injected groups. The results demonstrate that while on day one the percentages of Ova+ cells in HK and spleen are comparable, on day 5 and day 14 the values in spleen decline whereas those in HK remain high for up to 14 days. The data also show that in HK at all tested time points most of the Ova+ cells are MHCII+. The addition of 50 μg CpGs to the Ova, a dose that previously has been shown to upregulate expression of immune genes in both HK and spleen (Strandskog et al., 2008), did not seem to affect the accumulation of Ova+ cells in neither HK nor spleen. The experimental setup included five fish per group/time point; however, due to the large number of samples, for some of the groups only two samples could be analyzed which makes statistical analysis unreliable.

### *In vitro* uptake of dextran, Ova, and CpG ODNs by HK leukocytes

**Figure 2:**
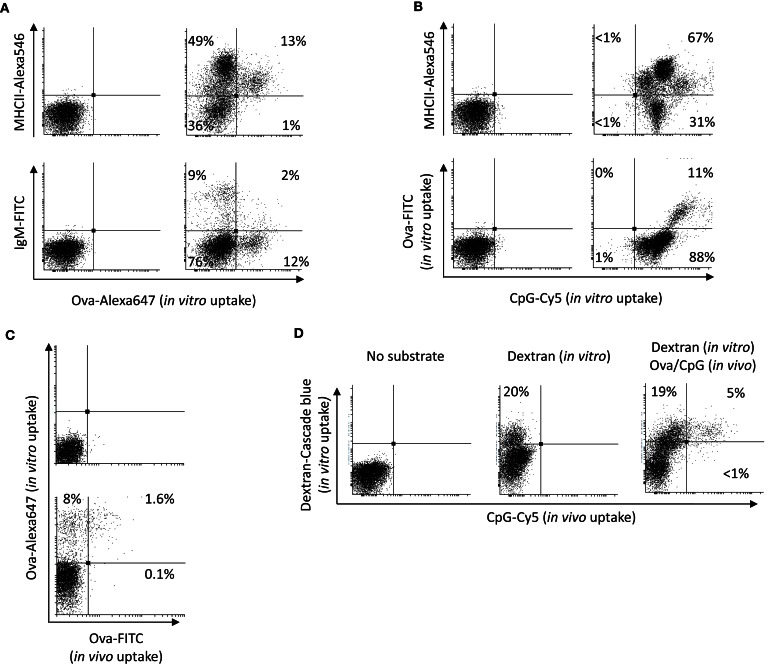
***In vitro* uptake of Ova CpGs and dextran by HK leukocytes**. **(A)** the vast majority of the cells that take up Ova *in vitro* are MHCII+/IgM−. HK cells were incubated *in vitro* with 10 μg/ml of Ova-FITC for 1 h prior to staining for surface MHCII and IgM and flow analysis. **(B)** In contrast with Ova, almost all of the HK leukocytes were able to take up significant levels of CpG ODNs. The cells that were able to endocytose Ova accumulated considerably more CpG ODNs as compared to the other cells. The HK leukocytes were incubated *in vitro* in the presence of Ova and 2 μM CpG-Cy5 ODNs for 1 h prior to staining and flow analysis. Both in **(A, B)**, the left dot plots show the non-stained controls. **(C)** The Ova-FITC+ cells found in HK after intra-abdominal injection endocytose Ova *in vitro*. The cells were incubated with 10 μg/ml of Ova-Alexa647 and the *in vivo* and the *in vitro* uptake of Ova was analyzed in the green and the far-red channels, respectively. The upper dot plot is a control with cells from PBS-injected fish, incubated without Ova-Alexa647. **(D)** CpG+ APCs found in the HK following an intra-abdominal administration exhibit high endocytic capacity *in vitro*. The cells were cultured in the presence of 150 μg/ml of dextran-Cascade blue conjugate prior to flow cytometry analysis. Representative plots from at least two experiments with cells from two individuals are shown.

The potential of HK leukocytes to take up Ova and CpGs was further analyzed *in vitro*. The results shown in Figure 2A demonstrate that, except for the higher percentage of leukocytes that were able to accumulate significant amounts of Ova (ranging between 9 and 20%), the *in vitro* experiment produced similar results as the *in vivo* trial. Namely, the substrate was accumulated at high levels exclusively by MHCII+ cells. The group of cells which were highly stained for surface MHCII and most of the IgM+ cells did not endocytose significant amounts of Ova. In contrast, almost all of the HK leukocytes accumulated large amounts of CpG ODNs (Figure 2B). Distinct cell populations endocytosed different levels of CpG ODNs. Of note, the cells with high capacity to endocytose Ova accumulated considerably more CpG ODNs as compared to the other cells types.

In order to investigate if the Ova-FITC+ cells detected in HK during the *in vivo* trial were still able to endocytose soluble Ag, HK cells from Ova-FITC-injected fish were incubated *in vitro* in the presence of 10 μg/ml of Ova-Alexa647 and the uptake of the *in vivo* and the *in vitro* endocytosed substrates were detected separately through the green and the far-red channels of the flow cytometer. The results are shown in Figure 2C and demonstrate that the Ova-FITC+ cells retained high capacity to endocytose the substrate *in vitro*. Due to the overlap between the emission spectra of Cy5 and the Alexa647 cells from fish injected with CpG-Cy5 ODNs could not be included in this experiment. Instead, dextran-Cascade blue conjugate was used to test the capacity of the CpG-Cy5+ cells found in HK during the *in vivo* trial to endocytose soluble substrate. As demonstrated in Figure 2D, the CpG+ cells had ability to endocytose high amounts of dextran *in vitro*, suggesting that these are still immature APCs.

### Adherent HK mononuclear phagocytes process Ova and accumulate the Ag within acidified endocytic compartments

**Figure 3:**
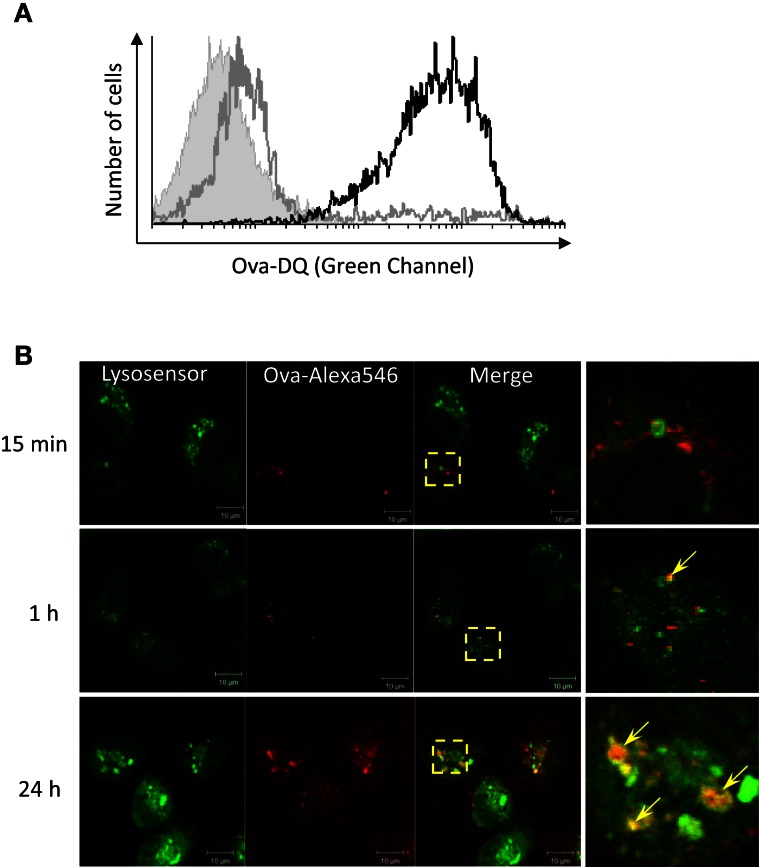
**Adherent HK mononuclear phagocytes hydrolyze Ova and accumulate the Ag within acidified endocytic compartments**. **(A)** Head kidney leukocytes were incubated for 4 h with 5 μg/ml of Ova-DQ. Non-adherent and adherent cells were harvested and analyzed separately with flow cytometry. Gray contour – non-adherent MHCII++ granulocytes, black contour – adherent MHCII+ cells. The filled contour shows a non-stained control. Ova-DQ fluorescence is detected in the green channel. Similar results were obtained with cells from two individuals. **(B)** Adherent head kidney mononuclear phagocytes attached to chambered coverglass slides were stained with LysoSensor Green (pKa 5.2) and incubated with Ova-Alexa647 for the indicated periods prior to live imaging using a confocal microscope. The arrows in the magnified overlap regions indicate accumulation of Ova within late endosome/lysosome compartments.

Ovalbumin-DQ is a self-quenched conjugate of Ova that exhibits bright green fluorescence upon proteolytic degradation. In order to investigate the ability of salmon HK APCs to process Ag, HK cells were incubated with Ova-DQ for 4 h followed by flow analysis. Non-adherent and adherent cells were harvested and analyzed separately after staining for surface MHCII. The results presented in Figure 3A show that unlike non-adherent MHCII++ granulocytes, adherent HK MHCII+ cells possess ability to effectively take up and process Ova.

The intracellular distribution of Ova was examined in adherent HK mononuclear phagocytes in which late endosomes and lysosomes were labeled with LysoSensor. As shown in Figure 3B, the endocytosed Ova was detectable in acidic vesicles within 1 h after addition of the Ag to the cell culture medium. The accumulation of Ova in LysoSensor+ vesicles was more pronounced after 24 h of incubation.

### Distinct HK cell populations are found based on surface MHCII and IgM expression, Ova uptake, and morphology

To further characterize the different HK leukocyte subpopulations cells were sorted using a FACSAria instrument according to their ability to endocytose Ova and the surface MHCII and IgM expression. The sorted samples were then analyzed using microscopy and RT-PCR.

**Figure 4:**
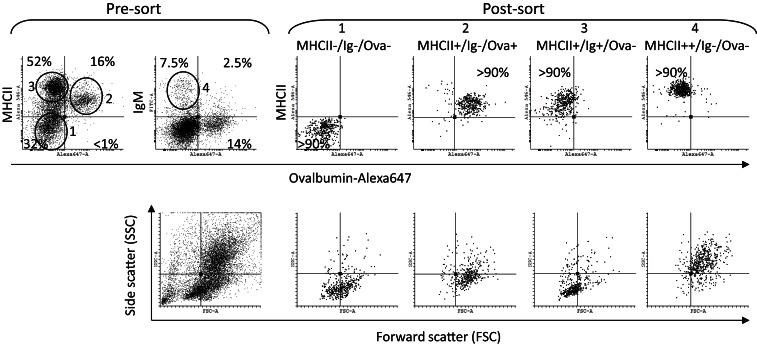
**Sorting of HK cells according to surface MHCII and IgM expression and Ova uptake**. The cells were incubated *in vitro* for 1 h with Ova-Alexa647 (10 μg/ml) double stained for surface MHCII and IgM and sorted using a FACSAria instrument. The pre-sort dot plots show the sorting gates setup. The post-sort analysis confirmed the sorting quality (>90%) for all of the populations and the morphology of the sorted cells was determined by the FSC (cell size) and the SSC (cell granularity) parameters, shown in the lower dot plots.

The pre-sort dot plots in Figure 4 show the setup of the gates. Unlike the Ova−/Ig+ cells which had typical lymphocyte morphology the Ova+/IgM+ cells were larger and granular and were excluded from the analysis as they may also have included macrophages which had bound IgM through their Fc receptors. As shown in the post-sort dot plots, nearly all of the IgM+ cells were also positive for surface MHCII. The post-sort analysis indicated that the purity of the sorted cells was at least 90% which was deemed high enough for further analyses. The side (SSC) versus the forward scatter (FSC) plots are included to show the morphology of the sorted cells. The MHCII−/Ig−/Ova−group included cells with variable size (FCS parameter) and low granularity (SSC). The MHCII+/Ig−/Ova+ group contained large cells with higher, but relatively modest granularity. The majority of the MHCII+/Ig+/Ova−cells were small and weakly granular whereas the MHCII++/Ig−/Ova−were large and highly granular, indicating that this group is composed of granulocytes.

**Figure 5:**
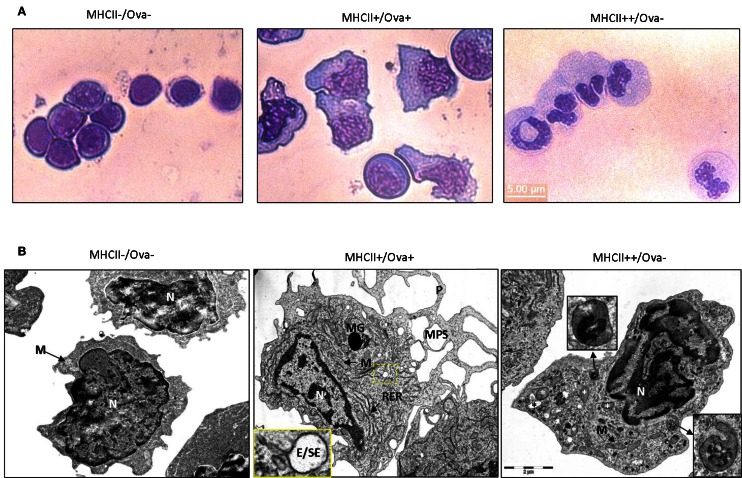
**Morphology of sorted HK cells**. **(A)** May Grünwald–Giemsa staining. **(B)** TEM images of sorted cells. Magnified regions are indicated with a dashed box and arrows. P, pseudopod; MPS, macropinosome; MG, melanin granule; M, mitochondrion. RER, rough endoplasmic reticulum; N, nucleus; E/SE, early/sorting endosome. Different types of granules/vesicles in MHCII+/Ova-cells are indicated with asterisks.

Light microscopy examination of May Grünwald–Giemsa stained sorted cells (Figure 5A), showed that the cells sorted in the MHCII−/Ova− gate displayed typical lymphocytic morphology with round nuclei, low cytoplasm versus nucleus ratio and intensely stained cytoplasm. The cells sorted in the MHCII+ gates had high cytoplasm/nucleus ratio. The Ova+ cells had mononuclear morphology with oval to bean-shaped nuclei and with a relatively densely stained cytoplasm. The MHCII++/Ova−granulocytes had polymorphic nuclei with finely granulated and a relatively weakly stained cytoplasm indicating that these are polymorphonuclear granulocytes. The ultrastructure of the sorted cells was examined with transmission electron microscopy (TEM) (Figure 5B). Except for the cell nuclei, a few small mitochondria and abundance of free ribosomes, no other clear structures could be observed in the MHCII−/Ova− cells. The MHCII+/Ova+ cells had long pseudopodia which upon fusion form macropinosomes (MPS) indicating that these cells perform macropinocytosis constitutively. The cells also had abundant mitochondria, extensive rough endoplasmic reticulum and, some of them, melanin granules. In addition, in some cells, early/sorting endosomes (E/SE) could be observed (magnified inset). Typically, these appear as vesicles with attached tubules into which the receptors such as those involved in the endocytosis of mannosylated substrates (i.e., the mannose receptor) are recycled toward the cells surface (Wileman et al., 1985; Jovic et al., 2010). Endocytosed proteins remain in the body of the E/SE and are transported toward degradation by lysosomal enzymes. The MHCII++/Ova−granulocytes contained many small and moderately sized vesicles (up to 400 nm) with varying density and round to elongated shape. Internal membranes with lamellar or vesicular shape were visible in some of these lysosome-like vesicles. Interestingly, one of the observed vesicles appeared to be in a process of microautophagy as suggested by the presence of an arm-like extension surrounding parts of the neighboring cytoplasm. No large pseudopodia, MPS, E/SE, and melanin granules were observed in these cells. Also, compared to the Ova+ cells these polymorphonuclear granulocytes contained fewer mitochondria and less RER.

### Different MHCII+ HK leukocyte subpopulations show distinct gene expression profiles

**Figure 6:**
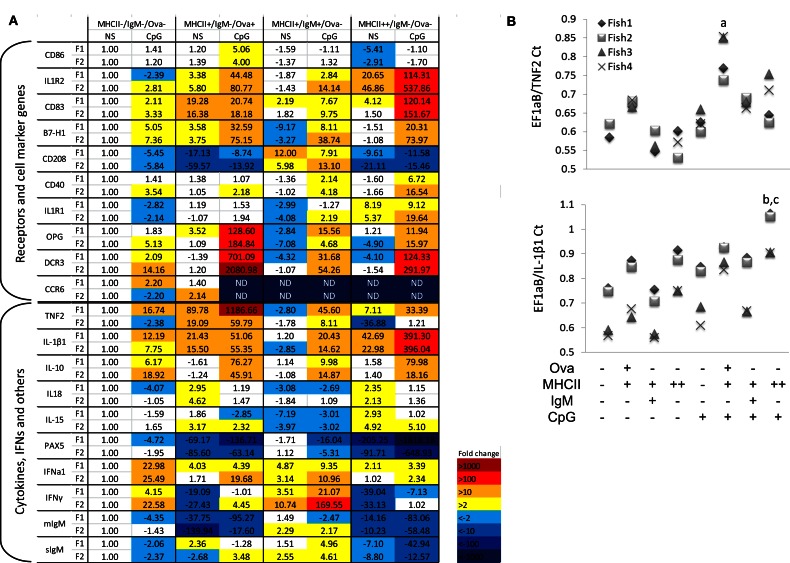
**Gene expression in sorted HK cells**. The cells were either left non-stimulated (NS) or treated with 2 μM CpG-B for 24 h prior to incubation with Ova-Alexa647 sorting as shown in Figure 3. Total RNA was isolated immediately after sorting and the gene expression was analyzed with Real-time PCR. **(A)** Gene expression is presented as fold difference values as compared to the non-stimulated MHCII−/Ig−/Ova−leukocytes. Data obtained with sorted cells from two individuals (F1 and F2) are shown in parallel. **(B)** The expression of TNF2 and IL-1β1 was analyzed in two additional individuals and is presented as a Ct ratio against the housekeeping gene. Note that in non-stimulated MHCII− cells from Fish 3 and 4 the TNF2 expression was undetectable whereas it was detected in all of the tested MHCII+/Ova+ cells. The TNF2 expression was analyzed with one-way ANOVA comparing the expression within the CpG-treated group (*n* = 4; a, *p* < 0.05 against all of the other cell types). The IL-β1 expression was analyzed with two-way ANOVA comparing the sorted samples across the non-stimulated and CpG-treated groups (*n* = 4; b, *p* < 0.05. compared to CpG-stimulated MHCII− and IgM+ cells; c, *p* < 0.05 compared to non-stimulated MHCII++ cells).

RNA samples from sorted cells were used to analyze gene expression in resting and in activated HK leukocytes subpopulations. CpG stimulation in higher vertebrate species directly activates DCs, macrophages, and B-cells, leading to upregulation of co-stimulatory molecules and proinflammatory cytokines and an increased capacity for antigen processing and presentation (Hartmann et al., 1999). Similarly, in Atlantic salmon, *in vitro* stimulation of adherent mononuclear phagocytes with CpGs for 24 h induced expression of proinflammatory cytokines and marker genes for mature APCs (Iliev et al., 2010). In the current study, the whole HK leukocyte population was stimulated with CpG-B for 24 h prior to sorting and gene expression analysis using RT-PCR. Among the leukocyte marker genes, the basal level of CD83, CD86, and B7-H1 was highest in the Ova+ cells (Figure 6A). However, the CpG treatment upregulated CD83 and CD40 most highly in the MHCII++/IgM−/Ova−cells. The expression of CD208 and IFN-γ was highest in IgM+ cells.

Interestingly, there was a contrast in the expression patterns of the proinflammatory cytokines TNF2 and IL-1β1. The former was most highly expressed in the Ova+ cells whereas both the basal and the CpG-upregulated IL-1β1 mRNA level were highest the MHCII++/Ig−/Ova−cells. The expression of the two cytokines was further confirmed in sorted cells from two additional fish, The TNF2 expression in non-stimulated MHCII− cells from fish 3 and fish 4 was not detectable making it impossible to calculate the “fold difference” expression of the cytokine in these samples. Nevertheless, as indicated by the ratio between the threshold cycle (Ct) values of the housekeeping gene and the cytokine, the MHCII+/Ova+ cells consistently expressed the highest levels of TNF2 mRNA, whereas the MHCII++/Ova−cells gave the highest levels of IL-1β1 mRNA when compared with other cell types from the same individual (Figure 6B). The MHCII++ granulocytes also expressed highest levels of IL1R1 and IL1R2 mRNA. On the other hand, the TNF receptor family members decoy receptor 3 (DCR3) and osteoprotegerin (OPG) were most highly upregulated in the Ova+ cells. In contrast with IL-1β1 and TNF2, the anti-inflammatory cytokine IL-10 was approximately equally upregulated by CpGs in Ova+ and MHCII++/IgM−/Ova−cells. IL-18 belongs to the IL-1 cytokine family and its expression was relatively high in Ova+ and in MHCII++/IgM−/Ova−; however, unlike IL1B1, it was downregulated by the CpGs. Like IL-18, IL-15 was not upregulated by CpGs and its expression was weakest in IgM+ cells. The CCR6 mRNA was detected only in the MHCII−/IgM−/Ova−and in the MHCII+/Ova+ cells and in the latter, the CpG treatment downregulated its mRNA to undetectable level.

PAX5 is a transcription factor involved in the early stages of B-cell development but its expression is suppressed during the late stages of plasma cell differentiation (Barberis et al., 1990) and a similar expression pattern for PAX5 has also been demonstrated in rainbow trout (Zwollo et al., 2005). In sorted cells, PAX5 was most highly expressed in the MHCII−/IgM−/Ova−and the IgM+ cells and the CpG stimulation downregulated its expression. The expression of the mIgM in resting cells paralleled that of PAX5 and the sIgM mRNA was induced by CpGs in IgM+ cells.

### Autophagy flux analysis

**Figure 7:**
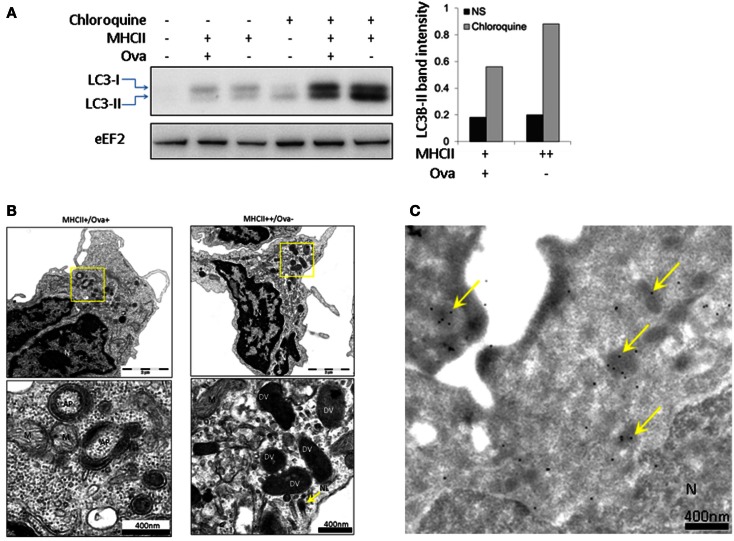
**Autophagy flux analysis in sorted HK subpopulations**. **(A)** HK cell were incubated with CHQ (5 μg/ml) for 24 h and sorted as described above. Protein samples from sorted cells were analyzed with WB using an antibody against LC3B. The histogram shows the intensity of the normalized LC3-II bands. eEF2 was used as a loading control. Similar results were obtained with samples from two individuals. **(B)** CHQ treatment induces formation of autophagosomes (AP) in MHCII+/Ova+ cells. Prior to sorting, the cells were treated with CHQ as in **(A)**. The autophagosomes appear as double-membrane vesicles which surround parts of the cytoplasm and are clearly distinguishable from other double-membrane structures, such as mitochondria (M). No specific AP formation could be observed in MHCII+/Ova− cells, however, the CHQ treatment enhanced the density of the electron-dense vesicles (DV), **(C)** LC3B is present in electron-dense vesicles in sorted MHCII++/Ova−granulocytes. Sorted cells were stained with the LC3B antibody and protein A labeled with gold particles. LC3-positive electron-dense vesicles are indicated with arrows.

Autophagy is a phylogenetically conserved mechanism through which eukaryotic cells degrade cytoplasmic material and whole organelles (Mizushima, 2007). IL-1β does not have a signal peptide and therefore it cannot be exported through the classical secretory pathway. It has recently been found that autophagy is implicated in the secretion of this cytokine (Klionsky et al., 2008; Dupont et al., 2011). The autophagy flux can be evaluated through Western blot (WB) analysis of the conversion of LC3B-I into LC3B-II, the latter of which migrates faster on PAGE. One potential problem with this analysis is the degradation of LC3B-II once the autophagosomes (AP) fuse with lysosomes (Klionsky et al., 2008). To block the lysosomal proteolysis and allow of LC3B-II to accumulate, CHQ was added to the cells 24 h prior to sorting and WB analysis using a commercial antibody. The representative image shown in Figure 7A demonstrates that following treatment with CHQ, the MHCII++/Ova−granulocytes accumulate higher amount of LC3B-II. However, TEM analysis showed that double-membrane vesicles which surround parts of the cytoplasm and appear to be AP could be observed only in the MHCII+/Ova+ cells but not in the MHCII++/Ova−leukocytes incubated with CHQ (Figure 7B). Nevertheless, the CHQ treatment increased the density of the vesicles found in the latter cells and immunogold staining/TEM showed that these vesicles were positive for LC3 suggesting that these might be involved in autophagy or autophagy-related processes (Figure 7C).

## Discussion

A major objective of the current paper has been to characterize salmon APCs in regards to their ability to take up soluble antigen and to migrate toward secondary lymphoid organs. The results demonstrate that there is a specific population of MHCII+ cells which are able to endocytose Ag in the periphery and which over a period of up to 14 days following the Ag administration in the abdominal cavity, accumulate predominately in salmon HK, but not in spleen. *In vitro*, these adherent cells accumulated Ova within late endosomes/lysosomes and were able to process Ova indicating that they are professional APCs.

The high capacity of APCs such as DCs to endocytose soluble Ags relies on receptor-mediated endocytosis and macropinocytosis (Sallusto et al., 1995). The former mechanism is mediated by receptors such as the mannose receptor expressed by macrophages and DCs and is necessary for the high-efficiency endocytosis of glycoproteins found on the surface of viruses, bacteria, and experimental substrates such as Ova and dextran (Sallusto et al., 1995; Burgdorf et al., 2006). On the other hand, the macropinocytosis is a process implicated in non-specific uptake of large amounts of extracellular fluid through membrane invaginations and lamellipodia (Lim and Gleeson, 2011). Both processes are downregulated upon activation of immature DCs by PAMPs or cytokines (Sallusto et al., 1995). In mammals, synthetic CpG ODNs are known to be potent vaccine adjuvants due to their potential to induce maturation of DCs (Askew et al., 2000; Klinman, 2003). This process is typically manifested by upregulation of surface MHCII and co-stimulatory molecules and downregulation of the Ag-uptake capacity trough inhibition of the receptor-mediated endocytosis and the macropinocytosis (Sallusto et al., 1995). It has previously been shown that *in vitro* stimulation of salmon mononuclear phagocytes with CpG-B upregulates a large array of immune genes, including markers for mature APCs, and it lowers the capacity of these cells to take up dextran and Ova (Iliev et al., 2010; Lagos et al., 2012). In the current study, injection with CpG ODNs did not seem to affect the migration of cells toward HK and spleen since the number of Ova+ cells in both organs did not differ significantly between the Ova and the Ova CpG-injected groups. Furthermore, the CpG treatment did not detectably upregulate the surface expression of MHCII and the HK CpG+ cells isolated following *in vivo* treatment were still able to endocytose significant amounts of dextran suggesting that these are still immature APCs. The CpG dose administered *in vivo* in the current study has been previously shown to induce expression of immune genes in salmon HK and spleen including IFN-γ and IL1β (Strandskog et al., 2008). However, the current data suggests that it may not be potent enough to trigger maturation of salmon APCs under these conditions.

While not very likely, it cannot be excluded that the substrates injected intra-abdominally may have diffused throughout the organism at high enough concentrations to be accumulated at detectable levels in HK and spleen cells *in situ*. The *in vitro* experiments indicated that this was not the case since a significantly higher percentage of HK leukocytes were able to accumulate high amounts of Ova and CpG ODNs *in vitro* as compared to the *in vivo* trial. This indicates that cells had taken up the substrate in the periphery prior to migrating toward the HK.

A previous study in which the expression of surface MHCII was analyzed in salmon HK and spleen leukocytes identified a population of granular cells which expressed relatively high levels of surface MHCII but had a low capacity to endocytose dextran and Ova (Iliev et al., 2011; Lagos et al., 2012). In the current study, the morphology of sorted cells, examined using May Grünwald–Giemsa staining and TEM indicate that these cells are most likely polymorphonuclear granulocytes. The relatively high MHCII expression on their surface and the upregulation of APC marker genes including CD83, CD40, and B7-H1 is somewhat surprising since in mammals granulocytes are typically, short-lived effector cells involved in clearance of bacterial and parasitic infections (Kobayashi et al., 2005). Nevertheless, studies have demonstrated that under specific conditions, murine neutrophils may upregulate MHCII and they can effectively present exogenous Ag to T-cells (Abi Abdallah et al., 2011). In addition, high expression of MHCII has been detected in seabream acidophilic granulocytes (functionally analogous to mammalian neutrophils) (Cuesta et al., 2006) and in zebrafish eosinophils (Wittamer et al., 2011) indicating that fish granulocytes may, potentially, be involved in antigen presentation.

In addition to exogenous substrate endocytosis, an alternative mechanism for delivery of antigens to intracellular MHCII compartments is autophagy. This is a process through which cytoplasmic constituents are delivered to lysosomes for degradation and it has been implicated in induction of immune response against intracellular parasites and in maintaining tolerance to autoantigens (Vyas et al., 2008; Klein et al., 2010). The relatively high level of conversion of LC3-II, in MHCII++ HK granulocytes indicates that these cells actively perform autophagy which may, potentially, be a source of peptides to be presented on MHCII molecules.

The CHQ treatment induced the appearance of typical AP in the MHCII+/Ova+ cell whereas in the MHCII++/Ova−granulocytes such double-membrane structures containing cytoplasmic material could not be observed. Nevertheless, the presence of LC3 and heterogeneous material in electron-dense vesicles found in the granulocytes indicates that these might be involved in autophagy, possibly microautophagy, or related processes such as LC3-associated phagocytosis. In this regard, LC3-II has been found in secretory granules of mast cells and autophagy has been shown to be crucial for mast cell degranulation (Ushio et al., 2011). Elucidating the involvement of autophagy in the function of the fish immune system will be a subject for additional, more focused studies.

The high migratory potential of immature DCs is conferred by the expression of various chemokine receptors. One of them, CCR6, is critical for the migration of murine DCs toward secondary lymphoid organs and upon maturation it is downregulated (Carramolino et al., 1999). Although, following CpG stimulation, the Ova+ cells did not upregulate surface expression of MHCII, the down regulation of CCR6 and the upregulation of CD86 indicate that they do have a potential to differentiate into mature APCs.

Interestingly, the upregulation of the proinflammatory cytokines TNF2 and IL-1β1 was cell type-dependent. While the former was most highly expressed in Ova+ cells, the latter was upregulated to the highest extent in the MHCII++/Ova−granulocytes. It could be argued that the upregulation of these cytokines might differ significantly based on the length of stimulation; however, it is notable that the level of expression of both cytokines in non-stimulated cells paralleled that in the CpG-stimulated samples. Considering this and the magnitude of the observed differences, the CpG-induced upregulation appears to reflect the general potential of the cells to express these cytokines. It has previously been shown that, in seabream, granulocytes are a major cell type that produces the IL-1β protein (Chaves-Pozo et al., 2004). Therefore, in the current study, the high expression of this cytokine’s mRNA in granulocytes indicates that these might be a key producer of IL-1β in salmon.

Unlike TNF which is expressed as a membrane bound protein, IL-1β lacks a secretory signal peptide and its mechanism of secretion has been debatable for a long time. It was shown in mammals as well as in seabream, that IL-1β was secreted within vesicles which appeared to be microvesicles (Pelegrin et al., 2004). However, more recently, it was suggested that a major route for secretion of IL-1β is through exosomes (Qu et al., 2007) – vesicles which are found in multivesicular bodies and which perform diverse immunomodulatory functions upon secretion (Denzer et al., 2000). Interestingly, it has been suggested that microautophagy, or a closely related mechanism, is involved in the formation of the exosomes (Buschow et al., 2005) and, it has been demonstrated that the IL-1β production and secretion depend on autophagy (Dupont et al., 2011; Harris et al., 2011). In view of this, the correlation between the high levels of LC3-II conversion and the IL-1β1 mRNA in the MHCII++ granulocytes suggests that these cells may be specialized for production of IL-1β.

The results from the current study show that the expression of TNF2 and IL-1β correlates very well with the expression of receptors for cytokines of these families. The granulocytes expressed exceptionally high levels of IL1R1 and IL1R2, the latter of which is a non-signaling decoy receptor for IL-1β, whereas the Ova+ cells upregulated highly DCR3 and OPG which are TNF family decoy receptors. The high upregulation of decoy receptors in cells that express high levels of cytokines for these receptors may serve to dampen excessive and potentially harmful effects of high concentrations of the cytokines in the microenvironment around the cells that secrete them.

Overall, the B-cells did not show particularly high expression of APC marker genes with the exception of CD208. In human, this molecule is specifically upregulated in mature DCs; however this trait does not seem to be phylogenetically conserved since in mouse it is present in other cells types (Salaun et al., 2003). The results shown in the current paper indicate that CD208 may serve as an excellent marker for salmon HK B-cells.

Compared to the other two MHCII+ cell types, the IgM+ cells expressed relatively low levels of proinflammatory cytokines. However, both the basal and the CpG-induced IFN-γ expression were very high in these cells. Although, in mammals, IFN-γ is predominately produced by NK and T-cells, certain subtypes of mammalian B lymphocytes are also known to express high levels of this cytokine (Harris et al., 2000).

It has been previously shown that trout HK harbors both early developing B-cells and antibody-secreting cells and serves as a major organ providing homing for mature plasma cells (Bromage et al., 2004; Zwollo et al., 2005). The CpG-induced downregulation of mIgM and PAX5 (a transcription factor expressed in the early/intermediate stages but not in the terminal stages of plasma cell differentiation) (Henderson and Calame, 1998) and the simultaneous upregulation of sIgM in IgM+ cells indicate that the stimulation induces differentiation of mature plasma cells. It is not very likely that CpG stimulation alone, without activation through the B-cell receptor could activate this process. However, provided these cells have already been activated through the B-cell receptor, the CpGs may contribute to the process of their terminal differentiation. In this regard, it has been previously shown that although Ag stimulation alone can activate B-cell proliferation, a second stimulus, provided by innate immune receptors, such as TLRs is necessary for induction of Ab secretion (Vos et al., 2000).

In summary, the presented data indicates that salmon HK may serve as a major secondary lymphoid organ to which APCs loaded with Ag in the peripheral organs migrate. The description of distinct gene expression profiles in different HK cell types will help define markers for further characterization of different APC types in salmon and other teleost species. The correlation between the high expression of IL-1β1 and the high autophagy flux in HK granulocytes suggests that these cells might be specialized toward production and secretion of IL-1β1. The presented data also lay a ground for further studies aimed at elucidating the significance of autophagy for the immune response in teleosts.

## Conflict of Interest Statement

The authors declare that the research was conducted in the absence of any commercial or financial relationships that could be construed as a potential conflict of interest.
